# Oxygen underpins the temperature-size relationship of protist plankton

**DOI:** 10.1128/msphere.00146-26

**Published:** 2026-08-31

**Authors:** Keisuke Inomura, Meng Gao, Moge Du, Curtis Deutsch

**Affiliations:** 1 Graduate School of Oceanography, University of Rhode Island 54083, Narragansett, Rhode Island, USA; 2 Department of Geosciences, Princeton University 140538, Princeton, New Jersey, USA; NC State University, Raleigh, North Carolina, USA

**Keywords:** growth rate, temperature, temperature-size relationship, protist, oxygen, model, size, plankton, cell flux model, cell volume

## Abstract

Temperature is known to affect organismal size, yet the underlying mechanism has been debated. Here, we develop a simple growth model of heterotrophic plankton and explain the temperature-size effect based on the demand and supply of oxygen. The model applies the growth rate-temperature relationship based on protein stability, capturing six data sets of a planktonic ciliate *Urotricha*. The model then computes the oxygen demand, based on the growth rate, which must be balanced by the diffusive uptake of oxygen. Because the smaller cell size favors oxygen uptake per cell volume, the predicted volume-temperature relationship is roughly the inversion of the growth rate-temperature relationship. With this inversive pattern, models are able to capture the data. This model-data consistency indicates that *Urotricha* may adjust their body size to meet the oxygen demands. Given that similar inversive relationships have been observed in other organisms, we hypothesize that oxygen demand and uptake are the key factors in constraining the volume of heterotrophic planktonic organisms.

**IMPORTANCE:**

The temperature-size relationship is a famous rule in physiological ecology based on common observations, but the mechanism underlying this rule has remained unclear. In this study, we develop a simple growth model of heterotrophic plankton, which explains the temperature-size relationship based on the demand and supply of oxygen. The model predicts temperature change will cause growth rate change; with higher growth rates, the increase of O_2_ demand causes a smaller cellular size. The model results also capture six datasets of *Urotricha* ciliates, suggesting that these organisms may adjust their body size to meet oxygen requirements. Given that similar patterns have been observed in other organisms, we hypothesize that oxygen demand and uptake are key factors constraining cell volume. Our study highlights oxygen as an intermediate factor underpinning temperature and cell volume, providing an explanation for the temperature-size relationship.

## INTRODUCTION

Temperature-size relationships are observed across microorganisms (1–8), yet the constraints behind that relationship remain unclear. This relationship provides useful information in modeling and prediction (9–11). Size is an important parameter related to chemical uptake by the cell that includes nutrients (12, 13), oxygen (14–16), carbon dioxide (17, 18), etc. Typically, small size is considered favorable for per-volume uptake of these molecules because it increases the surface-to-volume ratio of the cell (19–22). In particular, the uptake of small molecules that go through the cell membranes, such as oxygen and carbon dioxide, is strongly affected by the organismal size (14, 23). A recent study demonstrates a strong relationship between O_2_ uptake and body size across taxa, which indicates future shifts in body size due to changes in temperature across the ocean (24).

Among the diverse taxa showing this temperature-size relationship, *Urotricha* ciliates are representative cases. Experimental data show that their body size decreases with increasing temperature (25, 26). What causes this trend? We hypothesize that this question may be addressed by considering the balance of oxygen, specifically, the balance of cellular oxygen demand, oxygen uptake, and oxygen availability. Temperature-dependent changes in metabolism may be related to oxygen demand because oxygen is needed to fuel the metabolism (27, 28). Specifically, at moderate temperatures, growth rates increase with temperature (25, 29, 30), which may demand increased oxygen. Concurrently, oxygen solubility in water decreases (31, 32). Although the diffusivity of oxygen increases with temperature (33), this physical benefit may not fully compensate for the elevated metabolic demand. Therefore, cells may increase the oxygen uptake per volume by decreasing their size.

Reconciling these temperature effects on oxygen, we have developed a simple model of the temperature-size relationship (cell flux model of temperature-size relationships [CFM-TSR]) and tested it with the data of *Urotricha*. First, we applied a protein folding framework to represent the temperature-growth rate relationship. Then, based on that growth rate, we calculated the oxygen demand for the resultant cellular size. The model broadly captures the pattern of the temperature-size relationship in *Urotricha*, underpinning oxygen balance as a key factor in determining the cellular size at various temperatures.

## RESULTS AND DISCUSSION

**Fig 1:**
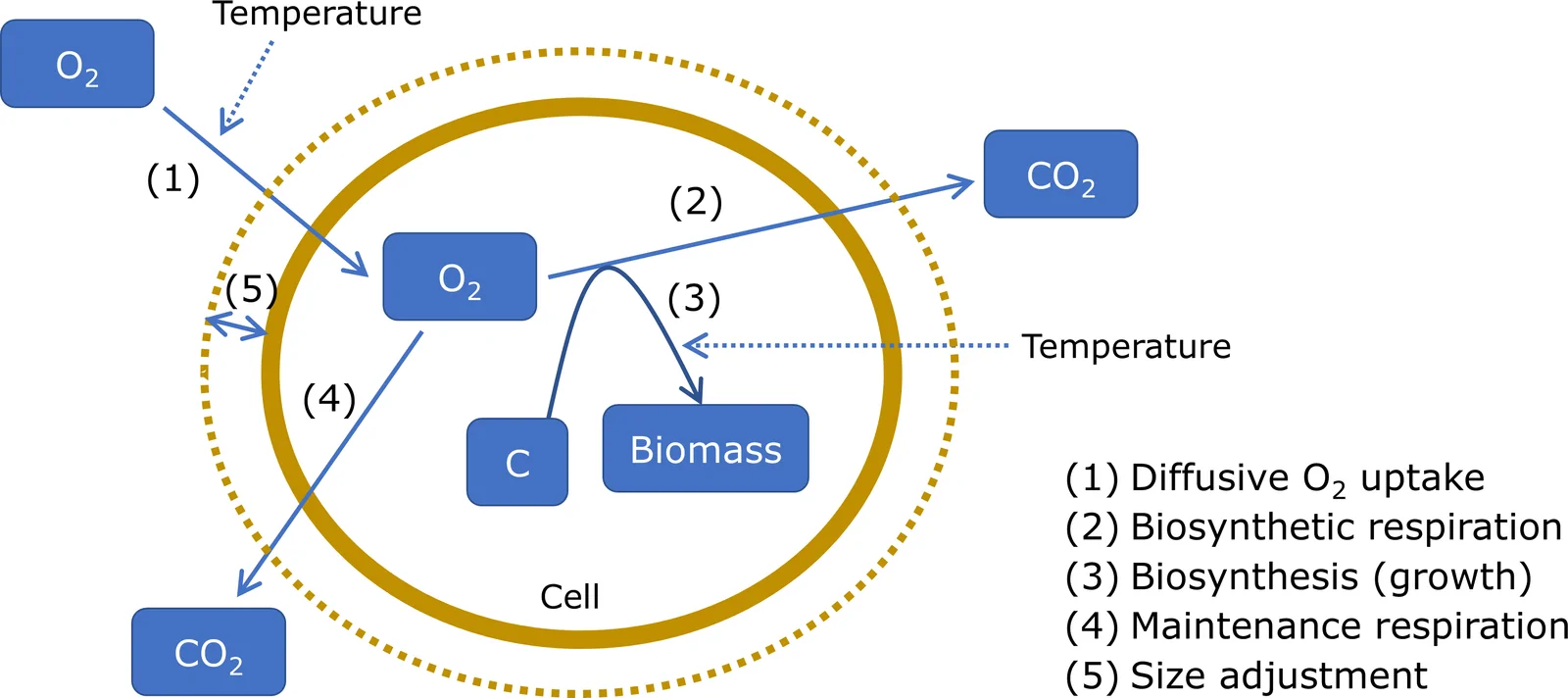
Schematic of the model: cell flux model of temperature-size relationships (CFM-TSR). One-way arrows denote the biochemical processes related to oxygen source (1, diffusive O_2_ uptake) and sink terms (2, biosynthetic respiration; 3, biosynthesis; and 4, maintenance respiration). Solid arrows are chemical fluxes and dotted arrows indicate tempreature dependencies.

CFM-TSR describes the relationship between the temperature, growth rate, and oxygen consumption (Fig. 1). The growth rate is a function of temperature based on protein stability (34–36). The oxygen consumption is then divided into two parts; one is biosynthetic respiration associated with growth, and the other is for cell maintenance respiration (Fig. 1; see equation 2 for parameterization). Growth rate and the oxygen demand have a positive correlation because a higher growth rate demands higher energy; thus, more oxygen is needed for respiration, which produces energy. The oxygen uptake is a function of size and temperature, and the model computes the cellular size that provides enough oxygen for cell growth. The detailed relationship is described in the method section.

**Fig 2:**
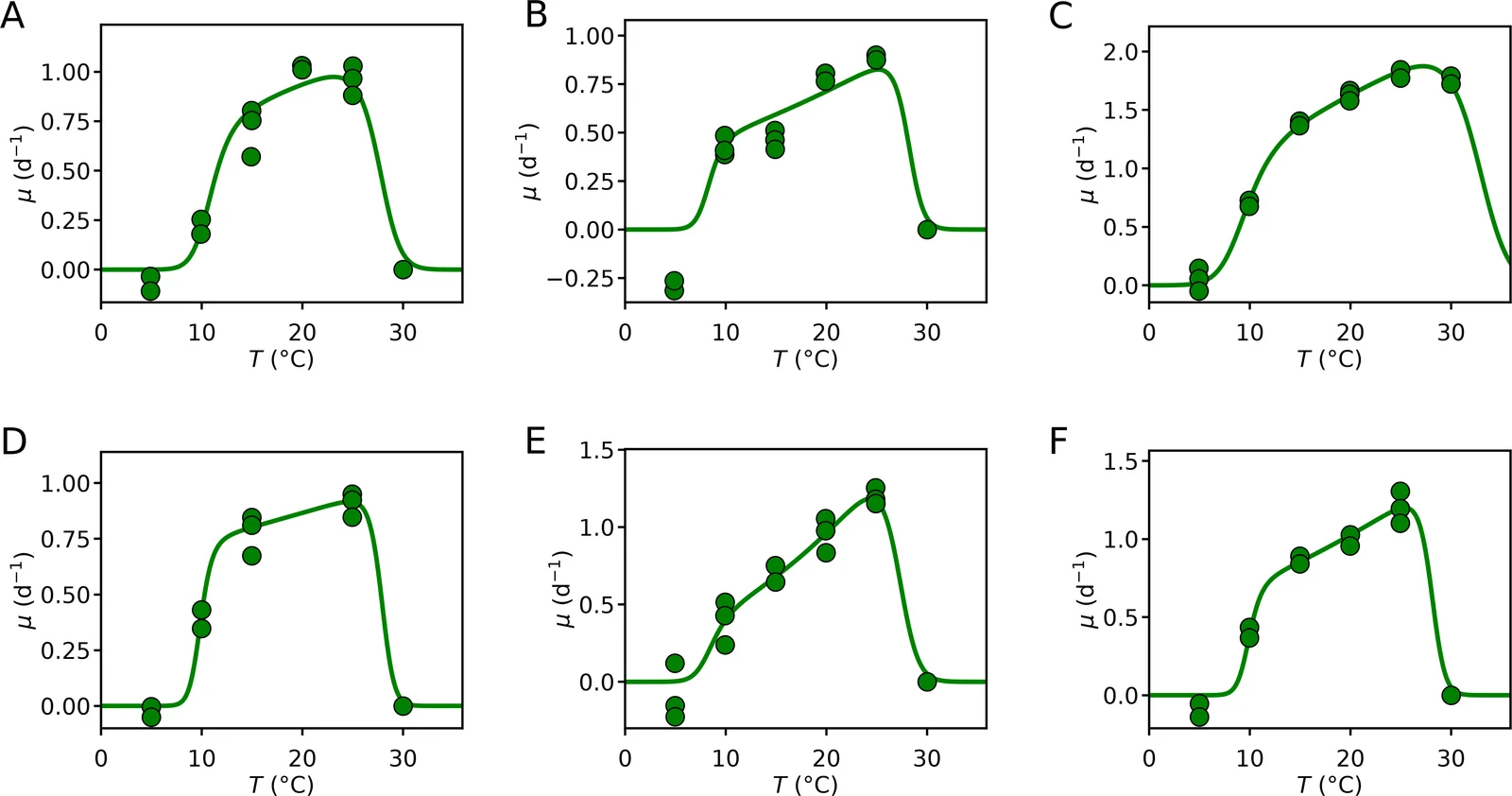
Model-data comparison of the temperature (*T*) effect on the growth rate (μ) based on protein stability of *Urotricha*. Scatter data in panels **A–F** are observations of six different isolates of *Urotricha* (25), and solid lines are the model outputs. Note that only one isolate (*Urotricha farcta*, panel **C**) was observed to grow at 30°C; thus, we manually added a point of μ = 0 at 30°C.

The model captures six observational data sets of the temperature dependences of the growth rate (Fig. 2). In this relationship, the model shows about three parts: increase, decline in slope, and decline. First, the growth rate starts increasing at 5°C–10°C. Second, the slope decreases, but the growth rate still increases. Finally, at 25°C–30°C, growth starts to decrease. Note that at 30°C, observational data show that cells do not grow except for one case (Fig. 2C); thus, we have manually added growth rate = 0 at that temperature and optimized the model. The predicted decline is sharp, which is consistent with the general trend when temperature exceeds the optimum (37, 38). There are some variations across the data, possibly due to the differences among strains (e.g., some show rather gradual increases than others). However, the model based on protein stability is able to capture the variation with different parameter sets.

**Fig 3:**
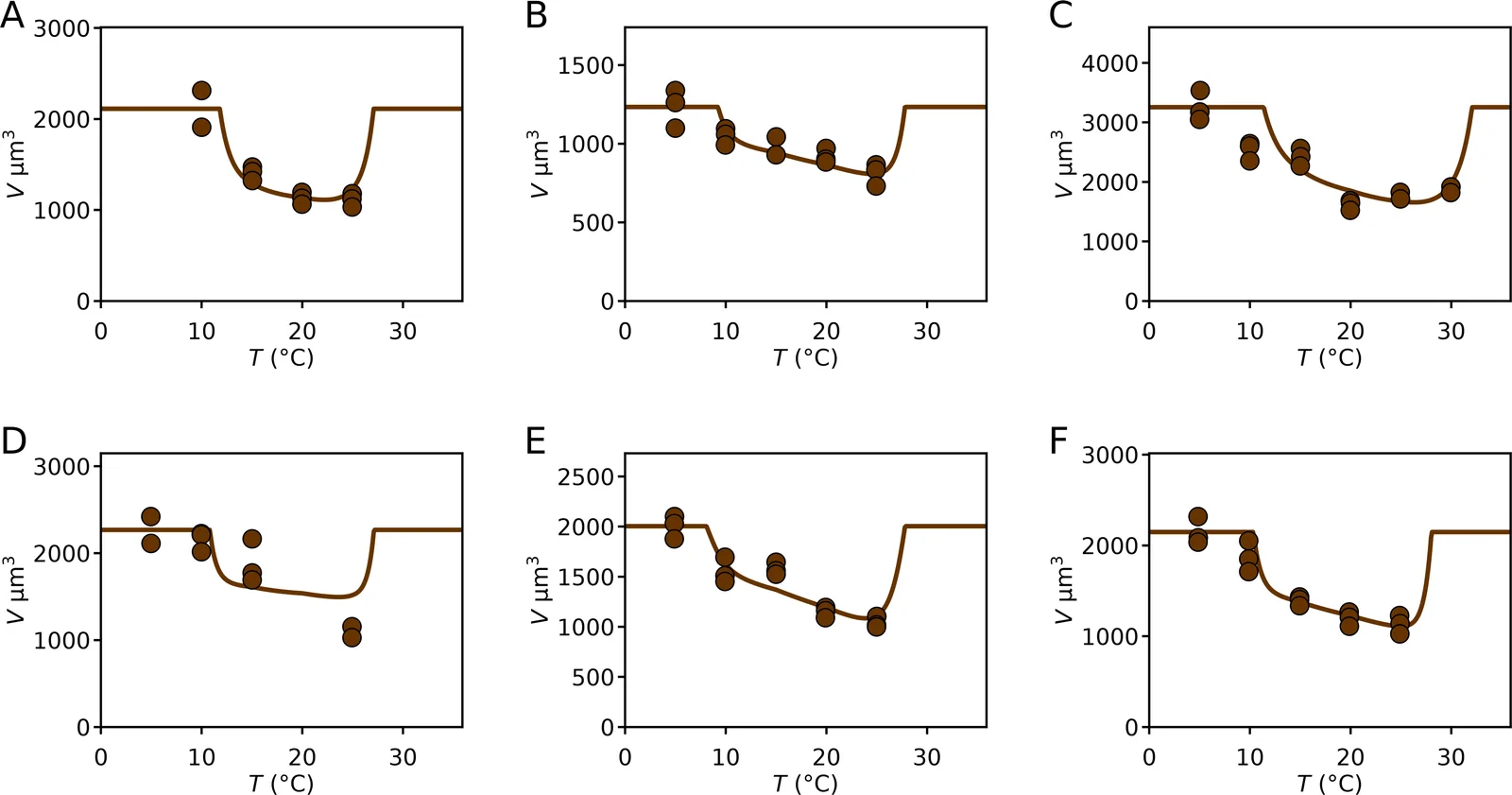
Model-data comparison of the temperature-size effect. Scatter data in panels **A–F** are observations of six different isolates of *Urotricha*, and solid lines are model outputs. Data are from identical isolates from Fig. 2 in the same order.

**Fig 4:**
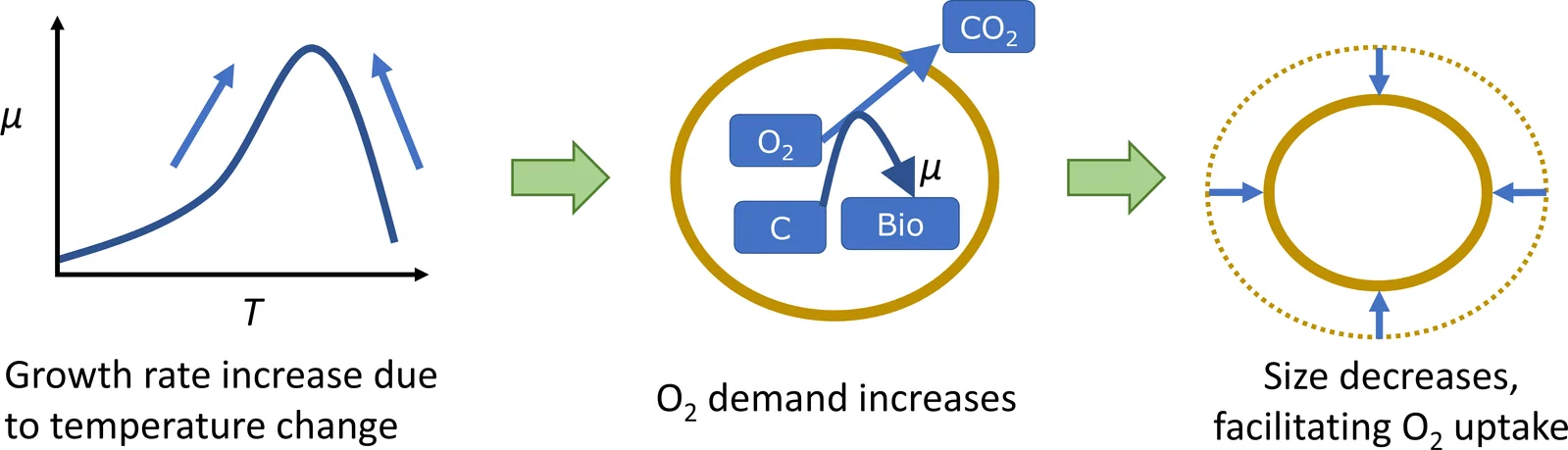
Summary from this study: physiological reasoning behind the temperature-size effect.

The simulated cell volume-temperature relationship is an overall inversion of the growth rate-temperature relationship (Fig. 3). Those results indicate that the model captures a plastic cell-volume response to temperature-dependent oxygen availability within a species-specific thermal range. As the growth rate increases, the requirement of oxygen increases. To maintain the growth rate, the oxygen demand must match the oxygen supply. Thus, as the growth rate and oxygen demand increase, cells decrease the volume because it decreases the surface-to-volume ratio and increases oxygen uptake per volume (Fig. 4). For a rising cell volume, unfortunately, there are only a few data points because cells did not grow at 30°C in most cases (25), which is a current limitation in fully validating the high-temperature simulations. Nevertheless, those predictions at temperatures around 30°C provide a valuable theoretical framework for understanding potential responses to extreme warming events. Future empirical studies and *in situ* observations focusing on heat-stress conditions (around 30°C) are highly recommended. Such data are important to further constrain the model and verify these predicted high-temperature results.

Our simple model allows high reproducibility of the data within current experimental temperature ranges, suggesting that oxygen uptake is an underlying mechanism for the size determination of *Urotricha*. DeLong (39), for example, experimentally showed in a predatory protist that biovolume production rates increased and cell size decreased with warming when prey supply was controlled. Our cellular model is consistent with this broader supply-demand framework but different in that we use oxygen as the resource linking temperature-dependent metabolic demand to cell-volume constraints. Our model is also different in that we vary supply of O_2_ based on the cellular size, following previous work (40–42).

Whereas the model shows that the growth rate variation and its associated oxygen demand are a large part of the story, maintenance respiration plays a secondary role. It is a respiration that occurs at zero growth rate to maintain the cellular state and to mobilize the cells (43, 44). It is possibly used for protein turnover as well. Because it adds some oxygen consumption, it decreases the cell volume. Its impact is especially pronounced where the growth rate is relatively small, and oxygen consumption is dominated by maintenance. The maintenance respiration increases the model-data congruency for some data, decreasing the relative effect of growth rate on oxygen consumption, indicating its impact on the cell size.

In the model, we also introduced maximum volume (Fig. 3, flat line), which improved the model representation of the data. Without imposing the maximum volume, where the growth rate is nearly zero, the model predicts significantly higher volume than observed. When maximum volume occurs, cells have excess oxygen because the cell volume is smaller than required based on the demand-consumption balance of oxygen. Our having to impose the maximum volume is conceptually consistent with the previous work (45), in which they observed apparently canalized (i.e., no plasticity) cells beyond a certain temperature range. They define the range that shows a negative relationship between the temperature and volume as “optimum,” and beyond that range, they occasionally observed a rather positive relationship between temperature and volume or a nearly constant volume (46). We show that the oxygen balance may explain such a positive relationship at temperatures higher than the optimum. At lower temperatures, a fixed volume at the maximum level provided a decent model-data fit. The presence of a cap in the volume is understandable because building cells costs resources (e.g., C, N, P, and trace metals), and when the temperature does not favor growth and molecular synthesis, having a large cell would be even more costly.

### Conclusions

We developed a model to interpret the temperature-size relationship in *Urotricha*, resolving the temperature effects on oxygen-related fluxes. The model captures the overall trends of the temperature-size relationship, underpinning the role of oxygen as an intermediate factor that holds this relationship. This relationship also indicates that oxygen is an ultimate growth-rate-limiting resource under nutrient repletion.

## MATERIALS AND METHODS

In this study, we relate temperature to cell volume through growth rate and oxygen uptake. The first step of the model is to compute the relationship between temperature and growth rate. To do that, we use the previously developed relationship based on protein stability (36):


(1) $$\mu =\frac{cT\exp\left(-\Delta H_{A}^{‡}/8.314T\right)}{1+\exp\left(-n\left\{\Delta H^{*}-18.1T+\Delta C_{P}[(T-373.6)-T\ln(T/385.2)]\right\}/8.314T\right)}$$

where *C* is a constant parameter, T is temperature, $\Delta H_{A}^{‡}$ is the enthalpy of activation, n is the number of amino acid residues in proteins, $\Delta H^{*}$ is the enthalpy change at the convergence temperature for enthalpy, and $\Delta C_{P}$ is the temperature-independent heat capacity change of the native and denatured state of proteins. This equation relates the protein stability with the growth rate because the growth and biosynthesis are mediated by enzymes. Conceptually, equation 1 combines two temperature-dependent effects. The numerator describes the Eyring-type increase in catalytic reaction rates with temperature for enzymes that remain active. The reciprocal of the denominator describes the probability that the controlling enzymes remain in their native, catalytically active state. Protein stability is therefore accounted for by the denominator: destabilization or denaturation at low and high temperatures lowers this probability and consequently reduces growth. The heat-capacity term $\Delta C_{P}$ captures this temperature-dependent destabilization. This formula has been demonstrated to reproduce various growth rate vs temperature relationships based on biochemical theories, which may not be reproduced by simple Arrhenius functions or *$Q_{10}$* formula (47).

Once we obtain the growth rate μ, we will relate it to the oxygen consumption rate $C_{O_{2}}$:


(2) $$C_{O_{2}}=(\mu +m)Q_{C}Y^{O_{2}:C}$$

where m is the maintenance constant, $Q_{C}$ is the cellular carbon (C) quota, and $Y_{}^{O_{2}:C}$ is a conversion term from the carbon consumption to oxygen consumption. The biosynthetic respiration and maintenance respiration (2 and 4 in Fig. 1, respectively) are represented by $\mu Q_{C}Y_{}^{O_{2}:C}$ and $mQ_{C}Y_{}^{O_{2}:C}$, respectively. For $Q_{C}$, we use a widely used $Q_{C}-V$ (volume) relationship of protist plankton (48):


(3) $$Q_{C}=aV^{b}$$

where a and b are constants (48). Under the steady state, the oxygen consumption $C_{O_{2}}$ must be satisfied by the oxygen uptake $V_{O_{2}}$, which can be expressed as


(4) $$V_{O_{2}}=4\pi RD_{O_{2}}[O_{2}]$$

with a spherical cell assumption (49, 50). Here, R is the cell radius, $D_{O_{2}}$ the is diffusion constant, and $\left[O_{2}\right]$ the is ambient oxygen concentration. The temperature dependencies of $D_{O_{2}}$ are simulated based on Walden’s rule (51):


(5) $$D_{O_{2}}=D_{O_{2}}^{ref}\frac{\eta_{ref}}{\eta }\frac{T}{T_{ref}}$$

where ref represents reference and η is the viscosity of solvent. For η values, we used the temperature-dependent viscosity of water (52). Assuming a steady state, where $C_{O_{2}}$ equals $V_{O_{2}}$, equations 2 to 4,4 can be solved for the cell volume:


(6) $$V={\left[\frac{{\left(48\pi^{2}\right)}^{1/3}F\frac{\eta_{ref}}{\eta }\frac{T}{T_{ref}}[O_{2}]}{a(\mu +m)}\right]}^{\frac{1}{3b-1}}$$

Here, $F=D_{O_{2}}^{ref}/Y_{}^{O_{2}:C}$. A more detailed derivation of equation 6 is in the Supplemental text. The $\left[O_{2}\right]\;\;$value was based on O_2_ saturation at a specific temperature at salinity of 35, based on (53). The relative change in $\left[O_{2}\right]$ with temperature is similar across salinity (53), and given that the value *F* is optimized as below, the effect of the salinity choice on the result in this study is small. Model parameters (Tables S1 and S2) are optimized with the Metropolis-Hastings algorithm (42) using data plotted in (Fig. 2 and 3). We first optimize parameters that impact μ in equation 1 with data in Fig. 2, and using these optimized parameters, we then optimize the remaining parameters *F* and *m* with the data plotted in Fig. 3.

## Data Availability

Code used for this study is freely available from GitHub/Zenodo at https://zenodo.org/records/21909328.

## SUPPLEMENTAL MATERIAL

Supplemental material msphere.00146-26-s0001.pdf Supplemental text; Tables S1 and S2.

ASM does not own the copyrights to Supplemental Material that may be linked to, or accessed through, an article. The authors have granted ASM a non-exclusive, world-wide license to publish the Supplemental Material files. Please contact the corresponding author directly for reuse.
