# Supplementary material for: Oxygen underpins the temperature-size relationship of protist plankton
Source: mSphere. 2026 Aug 31;11(9):e00146-26. doi: 10.1128/msphere.00146-26 (PMC13621864; doi:10.1128/msphere.00146-26)
Supplement: Supplementary file 1 [file msphere.00146-26-s0001.pdf]

Here we describe the deviation of Eq. 6 in the main text. Assuming a steady-state, where the oxygen consumption rate C_{O_2}(Eq. 2) equals the oxygen uptake V_{O_2}(Eq. 4), and Eq. S1 is obtained:

$$(\mu + m)Q_C Y^{O2:C} = 4\pi R D_{O2}[O_2] \qquad \text{Eq. S1}$$

Then a widely used Q_C-*V* (volume) relationship (Eq. 3) in the main text is used to substitute for Q_C in Eq. S1, yielding Eq. S2:

$$(\mu + m)aV^b Y^{O2:C} = 4\pi R D_{O2}[O_2] \qquad \text{Eq. S2}$$

Eq. S3 is the formula of sphere volume (*V*) to solve the radius of cells (*R*) in the equation (Eq. S3):

$$V = \frac{4}{3}\pi R^3,\ \ R = \left(\frac{3}{4\pi}V\right)^{\frac{1}{3}} \qquad \text{Eq. S3}$$

Eq. S3 is to substitute for *R* in Eq. S2, yielding Eq. S4:

$$(\mu + m)aV^b Y^{O2:C} = 4\pi\left(\frac{3}{4\pi}V\right)^{\frac{1}{3}} D_{O2}[O_2] \qquad \text{Eq. S4}$$

All terms containing *V* are moved to the left-hand side, yielding Eq. S5.:

$$V^{b-\frac{1}{3}} = \left[\frac{(48\pi^2)^{1/3} D_{O2}[O_2]}{a(\mu + m)Y^{O2:C}}\right] \qquad \text{Eq. S5}$$

Terms containing *b* are moved to the right side to obtain Eq. 6 in the main text, in which one side contains only *V*, with no other unknown variables.

$$V = \left[\frac{(48\pi^2)^{1/3} D_{O2}[O_2]}{a(\mu + m)Y^{O2:C}}\right]^{\frac{3}{3b-1}} \qquad \text{eq. S6}$$

Inserting Eq. 5 into Eq. S6 and defining $F = D_{O2}^{ref}/Y^{O2:C}$ will arrive at Eq. 6:

$$V = \left[\frac{(48\pi^2)^{1/3} F \frac{\eta_{ref}}{\eta}\frac{T}{T_{ref}}[O_2]}{a(\mu + m)}\right]^{\frac{1}{3b-1}}$$

Table S1. Parameter table

Parameters	Definition	Unit	Value
c	Constant parameter	dimensionless	Table S2
T	Temperature	K	273.15 – 308.15
$\Delta H_A^{\ddagger}$	Enthalpy of activation	$J\ (mol)^{-1}$	Table S2
n	The number of amino acid residues in proteins	dimensionless	Table S2
ΔH^{*}	Enthalpy change at the convergence temperature for enthalpy	$J\ (mol)^{-1}$	Table S2
ΔC_P	Temperature-independent heat capacity change of the native and denatured state of proteins	$J\ (mol\ K)^{-1}$	Table S2
μ	Growth rates	d^{-1}	Calculated by eq.1
m	Maintenance carbon loss	s^{-1}	Table S2
C_{O_2}	Oxygen consumption rate	d^{-1}	Calculated by eq.2
Q_C	Cellular carbon (C) quota	mol C	Calculated by eq.3
$Y^{O2:C}$	Conversion term from the carbon consumption to oxygen consumption	$mol\ O_2\ (mol\ C)^{-1}$	-
a	Constant	$mol\ C\ m^{-2.817}$	1437 (1)
b	Constant	dimensionless	0.939 (1)

D_{O2}	Diffusion constant	$m^2\ d^{-1}$	-
$[O_2]$	Ambient oxygen concentration	mol O_2 m^{-3}	Depending on temperature (2)
R	Cell radius	m	
V_{O_2}	Oxygen uptake	d^{-1}	Calculated by eq.4
V	Cell Volume	m^3	Calculated by eq.6
V_{max}	Maximum cell Volume	m^3	Average of V data at the temperature of 5 °C
D_{O2}^{ref}	Reference diffusion constant	$m^2\ d^{-1}$	-
η	Reference viscosity of solvent	$m^2\ s^{-1}$	Depending on temperature (3, 4)
η_{ref}	Reference viscosity of solvent	$m^2\ s^{-1}$	890.3 (value at T_{ref}) (3, 4)
T_{ref}	Reference temperature	K	298.15
F	$D_{O2}^{ref}/Y^{O2:C}$	mol C m^2 (mol O^2 d)$^{-1}$	Table S2

Table S2. Optimized model parameter values

	A	B	C	D	E	F
$\Delta H_A^{\ddagger}$	13694	23112	17056	8302	45820	22899
ΔC_P	65.6	64.8	67	65.3	64.4	65.4
ΔH^*	5340	5342	5347	5341	5341	5341
n	1227	1505	615	2017	1253	1878
c	0.898	32	6.22	0.0892	4.84×10^5	42.1
F	1.14×10^{-7}	1.13×10^{-7}	2.62×10^{-7}	1.13×10^{-7}	2.50×10^{-7}	1.72×10^{-7}
m	0.119	0.515	0.165	7.15×10^{-5}	1.28	0.490

References

1. Menden-Deuer S, Lessard EJ. 2000. Carbon to volume relationships for dinoflagellates, diatoms, and other protist plankton. *Limnol Oceanogr* 45:569–579.

2. Benson BB, Krause D. 1984. The concentration and isotopic fractionation of oxygen dissolved in freshwater and seawater in equilibrium with the atmosphere1. *Limnol Oceanogr* 29:620–632.

3. Korson L, Drost-Hansen W, Millero FJ. 1969. Viscosity of water at various temperatures. *J Phys Chem* 73:34–39.

4. Kestin J, Sokolov M, Wakeham WA. 1978. Viscosity of liquid water in the range −8 °C to 150 °C. *J Phys Chem Ref Data* 7:941–948.
